# Biomechanical analysis of different levels of constraint in TKA during daily activities

**DOI:** 10.1186/s42836-022-00157-0

**Published:** 2023-01-04

**Authors:** Gianluca Castellarin, Edoardo Bori, Laurence Rapallo, Silvia Pianigiani, Bernardo Innocenti

**Affiliations:** 1 II Unit Orthopaedic Department, Ospedale di Suzzara, 46100 Mantova, Italy; 2 BEAMS Department, Université Libre de Bruxelles, 1050 Bruxelles, Belgium; 3 Adler Ortho, Cormano, 20032 Milan, Italy

**Keywords:** TKA, levels of constraint, tibial stress, polyethylene stress, kinetics

## Abstract

**Background:**

Numerous total knee prosthetic implants are currently available on the orthopedic market, and this variety covers a set of different levels of constraint: among the various models available, a significant role is covered by mobile bearing cruciate-retaining design with an ultra-congruent insert, mobile bearing cruciate-retaining design, fixed-bearing posterior stabilized prosthesis and fixed-bearing constrained condylar knee. A biomechanical comparative study among them could therefore be helpful for the clinical decision-making process. This study aimed to compare the effect of these different levels of constraint in the knee biomechanics of a patient, in three different configurations representing the typical boundary conditions experienced by the knee joint during daily activities.

**Method:**

The investigation was performed via finite element analysis with a knee model based on an already published and validated one. Four different types of prosthesis designs were analyzed: two mobile-bearing models and two fixed-bearing models, each one having a different level of constraint. The different designs were incorporated in to the 3D finite element model of the lower leg and analyzed in three different configurations reproducing the landing and the taking-off phases occurring during the gait cycle and chair-rising. Implant kinetics (in terms of polyethylene contact areas and contact pressure), polyethylene and tibial bone stresses were calculated under three different loading conditions for each design.

**Results:**

The tibial stress distribution in the different regions of interest of the tibia remains relatively homogeneous regardless of the type of design used. The main relevant difference was observed between the mobile and fixed-bearing models, as the contact areas were significantly different between these models in the different loading conditions. As a consequence, significant changes in the stress distribution were observed at the interface between the prosthetic components, but no significant changes were noted on the tibial bone. Moreover, the different models exhibited a symmetrical medial and lateral distribution of the contact areas, which was not always common among all the currently available prostheses (*i.e.* medial pivot designs).

**Conclusion:**

The changes of the prosthetic implant did not induce a big variation of the stress distribution in the different regions of the tibial bone, while they significantly changed the distribution of stress at the interface between the prosthetic components.

**Supplementary Information:**

The online version contains supplementary material available at 10.1186/s42836-022-00157-0.

## Introduction

When the patient requires a total knee arthroplasty (TKA) but his posterior cruciate ligament (PCL) is healthy enough to ensure the stability of the knee joint, the surgeon could opt for the implant of a cruciate-retaining (CR) knee prosthesis. Those prostheses are categorized as non-constrained implants as the femoral and tibial components are not linked together and rely on the patient's native ligaments to guarantee the stability of the knee [1]. The CR TKA, furthermore, does not require slot resection on the distal femur to accommodate to the tibial post, thus preserving the femoral bone [1, 2]. Besides, good clinical outcomes are reported with CR TKA as it closely restores knee kinematics to the native knee, reducing patellar complications and shear forces [1, 3, 4]. The CR design, retaining more soft tissues than other implants, also preserves the proprioception of the patient, [3–5] thereby enhancing satisfaction levels. However, performing an appropriate soft tissue balancing is more challenging in the case of a CR knee as the PCL tension should be considered when balancing the flexion and extension gaps, which is even more difficult to achieve in case of larger knee deformities or PCL laxity [6].

**Fig. 1:**
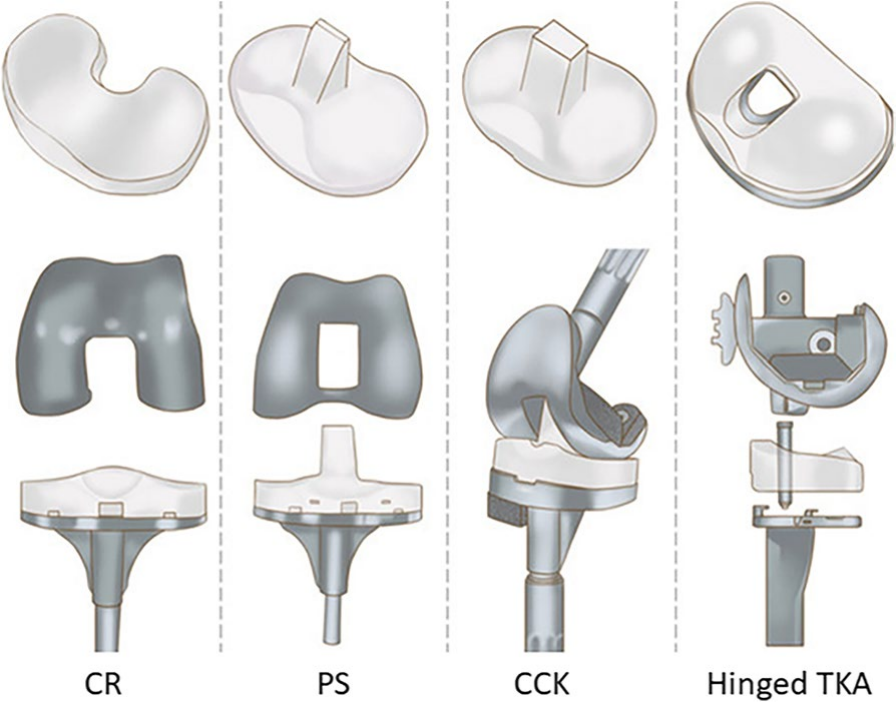
Different levels of constraint in TKA prostheses, from the less to the most constrained (from left to right)

The posterior stabilized (PS) designs, instead, involve PCL resection and incorporate a post-cam system to act as a substitute for the cruciate ligaments in anterior-posterior stability [4]. Those prostheses are classified as partially constrained, as they rely on the post-cam system to provide the missing constraint and therefore ensure the stability of the knee. The PS designs allow for the posterior movement of the femur, also known as femoral rollback. This factor is essential to achieving deep knee flexion, preventing, at the same time, the anterior translation of the femur on the tibia during knee flexion [4, 7]. PS TKA was therefore introduced to prevent posterior tibial subluxation and improve the range of motion with greater knee flexion [8, 9]. The main issue involved when using these prostheses lies in the contact occurring at the post-cam interface, which causes polyethylene wear at the level of the cam-mechanism and results in higher stress on the polyethylene insert itself [2, 10]. The constrained condylar knee (CCK) designs are considered as a semi-constrained prosthesis as they rely on a post-cam system like the PS designs, but this design presents a larger post and a deeper femoral box [11, 12]. While the post of the conventional PS is slightly rounded, the one of the CCK model is more rectangular with flat lateral and medial surfaces [13]. This design feature is intended to reduce internal-external (IE) rotation compared to the PS design, providing, at the same time, a higher varus and valgus stability from the increased level of constraint [10, 13, 14]. The geometry of the CCK post-cam system limits the flexion more than the one of the PS, and, as a consequence, the antero-posterior translation in high flexion is reduced as well. Those prostheses represent an alternative to more constrained prostheses for patients with medial and/or lateral collateral ligament insufficiency. These patients indeed require a more constrained articulation than a PS, but hinge prostheses are still considered too invasive for them [12, 13]. Therefore, CCK prostheses are considered more suitable as they involve less bone resection, especially for the distal femur [15]. Despite the higher level of constraint, severe flexion instability or collateral ligament deficiency could cause a dislocation of the knee and it is therefore mandatory to perform a hinged TKA [12]. Figure 1 shows the examples of the different levels of constraint in TKA prostheses addressed above.

Together with the different types of inserts listed above, a choice, in terms of insert-tibial tray constraint, is also available: These two components can be rigidly fixed or have a degree of freedom allowing for relative rotation. In mobile-bearing (MB) implants, indeed, the tibial insert can rotate upon the tibial component while in fixed-bearing (FB) implants the tibial insert is fixed firmly to the metal tibial tray. MB TKA is thus designed with a rotating platform, providing higher conformity of the different components' articular surfaces [16]. Thanks to this design feature, the mobile bearing inserts aim to provide more physiological kinematics and kinetics of the prosthesis, correcting the small tibial rotational misalignment and furthermore minimizing the polyethylene wear by decreasing relative displacements and stress [12, 17, 18]. In this category of mobile inserts, a further distinction between standard and ultra-congruent inserts can also be made. Compared to the standard ones, ultra-congruent inserts are characterized by an increased anterior buildup, providing a more conforming articular surface that better matches the radius of the femoral component [19]. These inserts are able to guarantee a good anterior-posterior stability without the need of a metal cam [20, 21], and with the even increased congruency, contact stress peaks are theoretically avoided, providing better stress-forces distribution [21].

With all the different options provided, the surgeon has then to choose the appropriate combination from the wide range of existing implant components, taking into consideration all their relative implications on the short- and long-term survival of the implant. In order to provide a clearer overview of the outcomes expected from the different possibilities, and thus decide which could represent the ideal one, this study took into consideration and biomechanically analyzed different prosthesis configurations from the same family of products (with the aim of avoiding eventual influences induced by the changes in brand-related design, thus being able to compare the level of constraint only). The implants analyzed present different levels of constraint and, as a consequence, each implant provides a different stability in the TKA and distinct interactions are observed between the bone and the prosthesis [22].

The purpose of this study was therefore to compare the effect of these four prosthetic implants with their different levels of constraint in the knee biomechanics of a patient, simulating three different configurations representing the typical boundary conditions experienced by the knee joint during daily activities.

Finite element analysis is a technique that involves the use of a numerical solver to implement an input model and to perform a virtual simulation, then providing the consequent mechanical outputs: it represents thus an optimal tool to perform this type of analysis. The possibility offered by this approach is to guarantee the application of a same exact set of boundary conditions on different models, and this represents one of the fundamental features that contribute to the wide adoption of this technique in the research and industrial biomechanical fields [23].

## Materials and methods

### Geometry

**Fig. 2:**
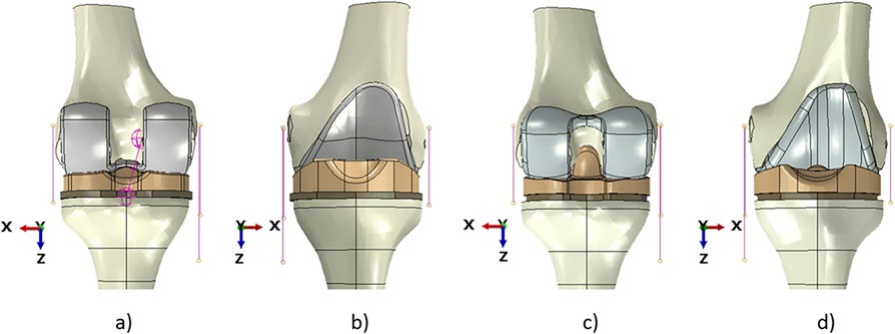
Full knee models with different prostheses implanted; (**a**) MB CR prosthesis, posterior view; (**b**) MB CR prosthesis, anterior view; (**c**) FB PS prosthesis, posterior view; (**d**) FB PS, anterior view

The finite element model developed for this study was based on a previously validated and published knee finite element model [24], and it includes the features reported hereafter. Three-dimensional tibial and femoral bone models were extracted from computer tomography images of left Sawbones composite tibia and femur, size medium [25]. These geometries were then accordingly partitioned in cortical and cancellous bone [25, 26]. The lateral collateral ligament (LCL) and the medial collateral ligament (MCL) were incorporated into all the models [27] as pre-strained beams with a specific cross-section [9, 24, 26–28] (Table 1), with the medial one modeled as two distinct parts (anterior medial collateral ligament (aMCL) and posterior medial collateral ligament (pMCL)) according to previous studies [27]. The insertion points of each collateral ligament (LCL, aMCL and pMCL) were determined by following the literature [29]. Finally, the posterior cruciate ligament (PCL) was then incorporated only in the models involving the MB CR implants, with the same approach as the other ligaments involved. The family of products analyzed included four prosthesis designs: two different GENUS MB CR models (one with a standard insert and one with an ultra-congruent one), an FB PS, and an FB CCK one. The CAD files of the different prosthetic components (femoral component, tibial insert and tibial tray), all being left sides, were provided by the company Adler Ortho (Cormano, Milan, Italy) and were virtually implanted on the knee model by following the instructions provided by the manufacturer. All the models were thus implanted according to referenced surgical techniques [9, 25, 27], using the press-fit fixation approach and following the mechanical alignment of the knee [27]. The two GENUS MB CR models have the same femoral and tibial components, with only the insert being different. More specifically, a standard model and ultra-congruent ones were selected and analyzed. The same tibial component was used in both FB designs. It is important to note that the FB and MB tibial components present a slightly different stem design, with one of FB being longer and larger. For the four prostheses, the femoral components and the tibial inserts were of size 6 while the tibial components were of size 5 to closely fit the geometries of the bones used. Fig. 2 illustrates TKA designs with one of two MB models (MB CR knee) and one of the two FB models (FB PS knee).

**Table 1:** Pre-strain of the collateral ligaments

Ligament	Pre-strain ε_**r**_
LCL	0.08
MCL	0.04

### Material models and properties

According to previous studies [9, 24, 25, 27–29], linear elasticity was used for all the material models considered in this study. The cortical bone was considered linear transversely isotropic (with the principal axis corresponding to the anatomical axis of the bone) whereas the cancellous bone was considered linear elastic isotropic [9, 25, 27, 29–31].

The LCL and MCL were considered isotropic and a validated pre-strain modeling approach was followed [9, 24, 25, 27, 28] (Table 1). The PCL was modeled as a spring with a stiffness of 350 N/mm [23].

The femoral component, as well as the tibial component, were given the properties of cobalt-chromium (CoCr), while for the tibial insert ultra-high-molecular-weight polyethylene (UHMWP) was used. All these materials were assumed to be homogeneous and isotropic [9, 15, 25, 28, 32] and the properties used to model them are reported in Table 2.

**Table 2:** Material properties of the knee model; 1 = Mediolateral Axis of the Bone; 2 = Anterior-Posterior Axis of the Bone; 3 = Anatomical Axis of the Bone

Material	Material model	Young's modulus (MPa)	Poisson's ratio
Cortical bone	Transversely isotropic	E_1_ = 11,500	ν_23_ = 0.31
		E_2_ = 11,500	ν_13_ = 0.31
		E_3_ = 17,000	ν_12_ = 0.58
Cancellous bone	Elastic isotropic	2130	0.31
CoCr	Elastic isotropic	240	0.30
UHMWP	Elastic isotropic	0.70	0.40
LCL	Elastic isotropic	345	0.45
MCL	Elastic isotropic	332	0.45

Tie contacts were implemented between the femoral bone and the relative prosthetic component, while general contact was adopted to simulate the interface between the tibial bone and the tibial component. A coefficient of friction of 0.04 was considered for the interaction between all the prosthetic components (between the femoral component and the tibial insert and between the tibial insert and the tibial component) [12, 26, 27, 32].

### Analyzed configurations

**Fig. 3:**
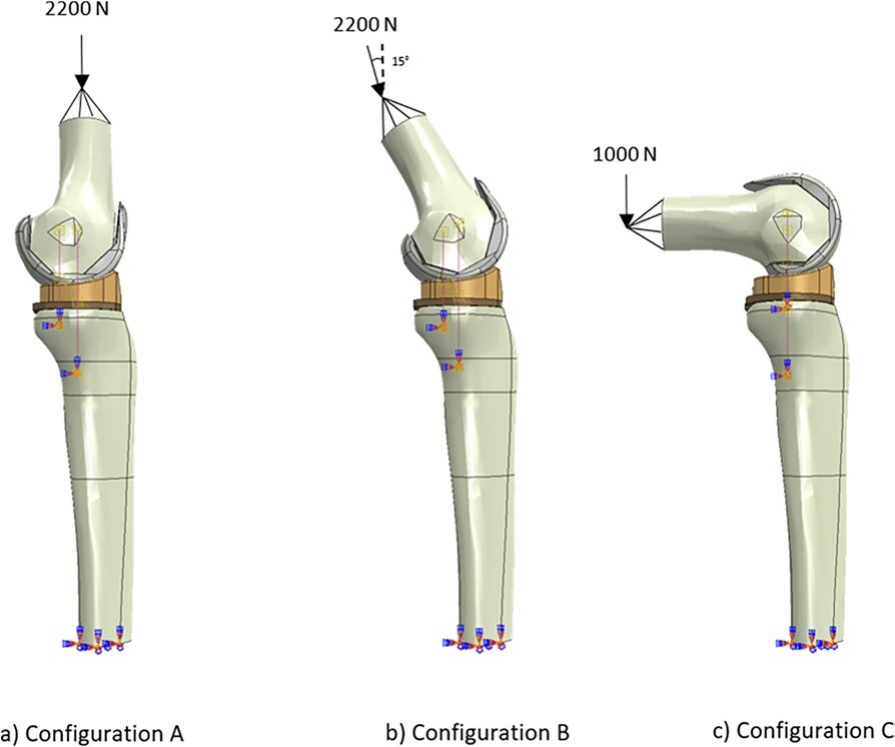
Illustration of the forces applied to the MB CR model in the three analyzed configurations

Three configurations were defined according to previous studies [9, 12, 15, 30, 33–37] and are illustrated in Fig. 3 (the MB CR model is shown, but the same load conditions are applied to all four models). They represent the solicitations taken by the knee joint during the most frequent activities of daily life, such as walking and standing up from sitting. In all configurations, the distal tibial extremity was fully constrained, and the proximal femoral surface was coupled with a reference point to apply the different loads involved.

In details:

- Configuration A reproduced the landing phase occurring during the gait cycle (Fig. 3a) and is defined with a flexion angle of 0° and a vertical load of 2200 N, applied to the reference point coupled with the proximal surface of the femur and directed along its mechanical axis. This force replicates the maximal knee axial force achieved during gait [14], corresponding to about 3.1 times of 70 kg body weight (as already implemented in previous studies [24, 26, 28, 29, 34, 38].
- Configuration B represented the taking-off phase occurring during the gait cycle and is defined with a flexion angle of 30° and a vertical axial load of 2200 N, applied to the reference point coupled with the proximal surface of the femur and with an inclination of 15° with respect to the direction of the tibial longitudinal axis (Fig. 3b). This maximal axial force, already used in the first configuration, was implemented also in this one in order to consider the eventual worst-case scenario.
- Configuration C is defined with an angle of 90° of flexion and a vertical load of 1000 N, applied to the reference point coupled with the proximal surface of the femur and directed along the tibial mechanical axis (Fig. 3c). This configuration aims to investigate the sit-to-stand transfer from a chair. In this configuration, it is possible to note that the force required is not as high as in the two previous configurations. This is due to the fact that the "sit-to-stand" task is usually performed with the help of the hands which therefore help in unloading the knee joint during the movement [35].

Regarding the femur, any form of rotation or displacement (internal-external rotation, varus-valgus rotation, antero-posterior translation, medio-lateral translation, inferior-superior translation) was allowed except for the flexion-extension, in order to keep each model in the studied configuration with the appropriate flexion angle.

In order to simulate the different behaviors of fixed and mobile bearing prostheses, different constraints were applied to the inserts. For both fixed-bearing models, the relative motion between the insert and the tibial tray was rigidly constrained via a tied contact; for both mobile bearing models, instead, the insert was allowed to rotate about the longitudinal axis thanks to the relative degree of freedom left unconstrained.

In all the analyzed configurations, the tibia and all the relative collateral ligaments attachment points (aMCL, pMCL and LCL) were distally fixed [9, 25, 28–30, 39].

### Finite element analysis & outputs

All the parts of the models were meshed using linear tetrahedral elements, with the element sizes of each part being between 1 and 5 mm. This was chosen based on a convergence test performed to verify the mesh quality for every region of the model. ABAQUS/ Standard version 2019 (Dassault Systèmes) was used to perform all the finite element simulations.

### Regions of interest and outputs

**Fig. 4:**
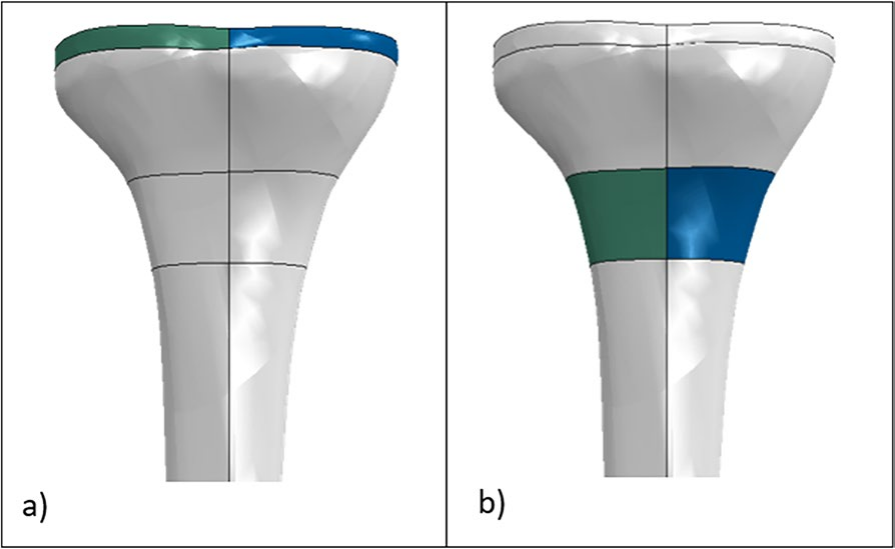
Regions of interest in the tibia (green and blue represent respectively the lateral and medial regions), posterior view; (**a**) proximal tibia regions; (**b**) distal tibia regions

Four regions of interest were defined to study the tibial bone stress:

- The medial and lateral proximal zones: two regions close to the tibial tray with a thickness of 5 mm (Fig. 4a);
- The medial and lateral distal zones: two regions 30 mm from the tibial cut surface with a thickness of 20 mm (Fig. 4b).

The first two regions were selected to compare the tibial stress induced by the different types of prostheses in the region closest to the cut, while the distal two aimed to provide a more global overview of the variation of the stress distribution (on both medial and lateral sides) along the tibial bone. This decision was taken in agreement with the literature [25]. For all the models, the medial and lateral average von Mises tibial stress in the cortical bone alone and in both cortical and cancellous bones was investigated in all the proximal and distal zones.

**Fig. 5:**
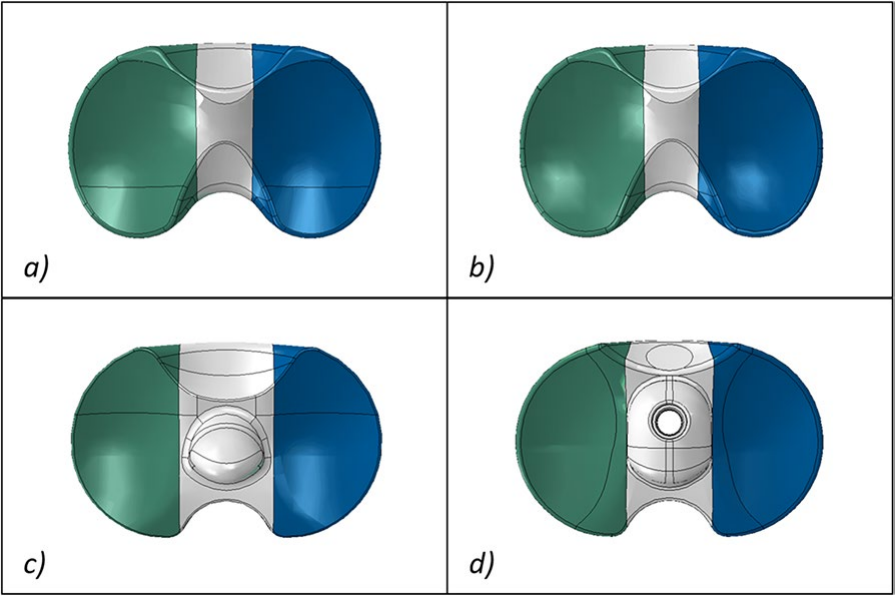
Regions of interest analyzed for the inserts (green and blue represent respectively the lateral and medial regions); (**a**) MB CR insert; (**b**) MB CR ultra congruent insert; (**c**) FB PS insert, and (**d**) FB CCK insert

In order to conduct a more in-depth analysis of the different outcomes, the tibial prosthesis inserts were divided into three parts: medial, lateral and middle ones (Fig. 5). Medial and lateral contact areas, contact pressures as well as von Mises stresses were the parameters of interest, and they were then compared among the four models for the three addressed configurations.

## Results

**Fig. 6:**
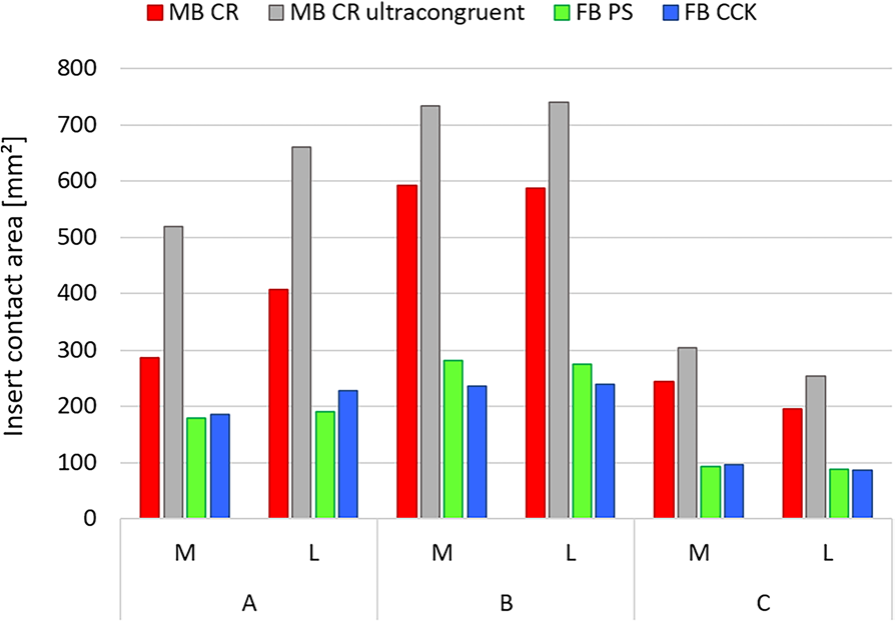
Medial (M) and lateral (L) insert contact area in the three studied configurations

**Fig. 7:**
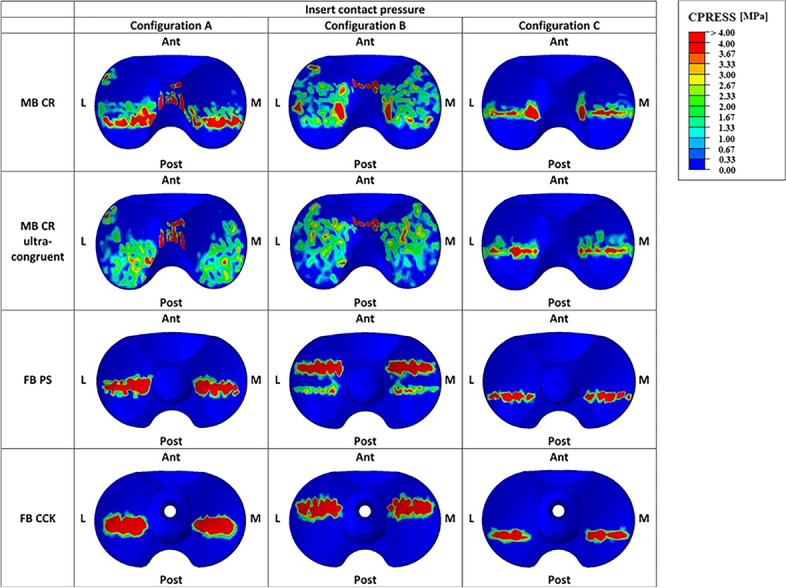
Qualitative overview of the insert contact pressure in the three studied configurations (M=medial, L=lateral, Ant=anterior, Post=posterior)

**Fig. 8:**
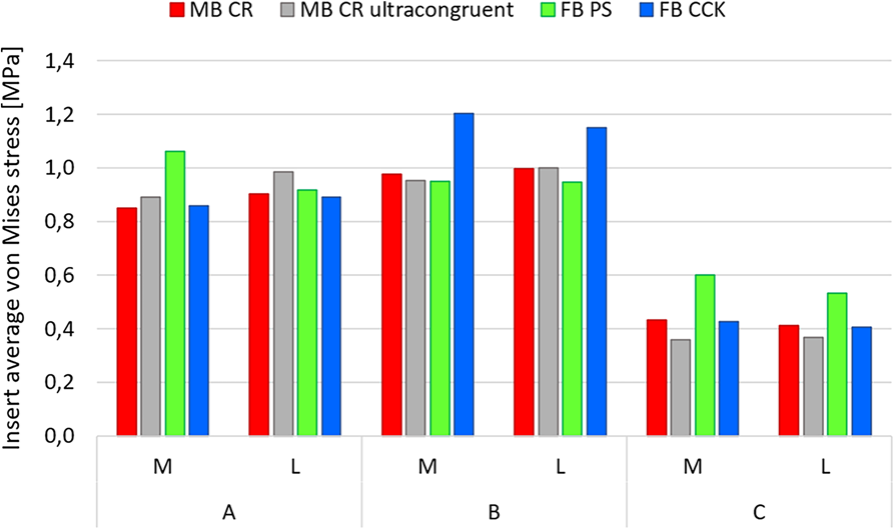
Medial (M) and lateral (L) average polyethylene von Mises stresses for the different models in the three studied configurations

Figures 6 and 7 illustrate respectively the contact areas and contact pressures in the three studied configurations for all four insert designs. These results show that the MB CR ultracongruent design presented the highest congruency, with its contact area value being almost 3 times higher than the one of the fixed-bearing inserts. Figure 8 reports the quantitative values of the average von Mises stresses in the medial and lateral regions of interest of the polyethylene insert, for the three different configurations for each studied model. The analysis showed relatively low von Mises stresses for every design, with the average medial and lateral values being below 1.2 MPa. The FB CCK yielded higher stresses in configuration B compared to the other insert models, while FB PS had higher values for the medial side in configuration A and for both sides in configuration C.

**Fig. 9:**
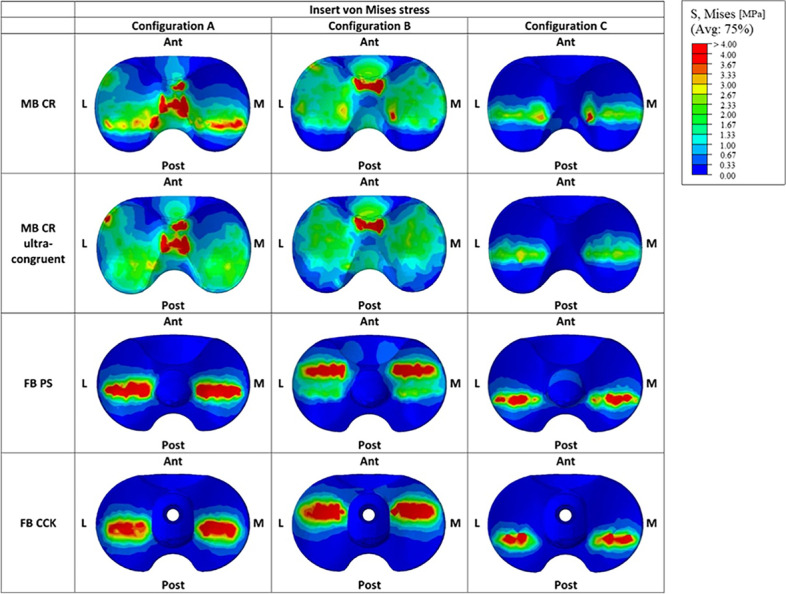
Qualitative overview of the polyethylene von Mises stresses for all the considered models in the three studied configurations. (M = medial, L = lateral, Ant = anterior, Post = posterior)

Figure 9 reports the qualitative overview of the average polyethylene von Mises stresses in the three studied configurations for each model. This graph exhibits a similar stress distribution for both MB designs and for both FB designs. Moreover, the stress appeared to be symmetrically distributed on both medial and lateral parts of the insert for all models (Figs. 8 and 9). The ultra-congruent insert was more compliant with the femoral component in its anterior part, presenting, therefore, a higher contact area when the contact occurred in this part if compared to when it happened in the center of the insert. It is thus possible to see, with Fig. 6, that the total contact area found for configuration A (considering both medial and lateral contribution) of the MB CR ultra-congruent insert increases of approximately 25% in the configuration B and decreases of almost 50 % in the configuration C. For both FB inserts, the stress was distributed more anteriorly in the configuration B while it was located more posteriorly in the configuration C.

**Fig. 10:**
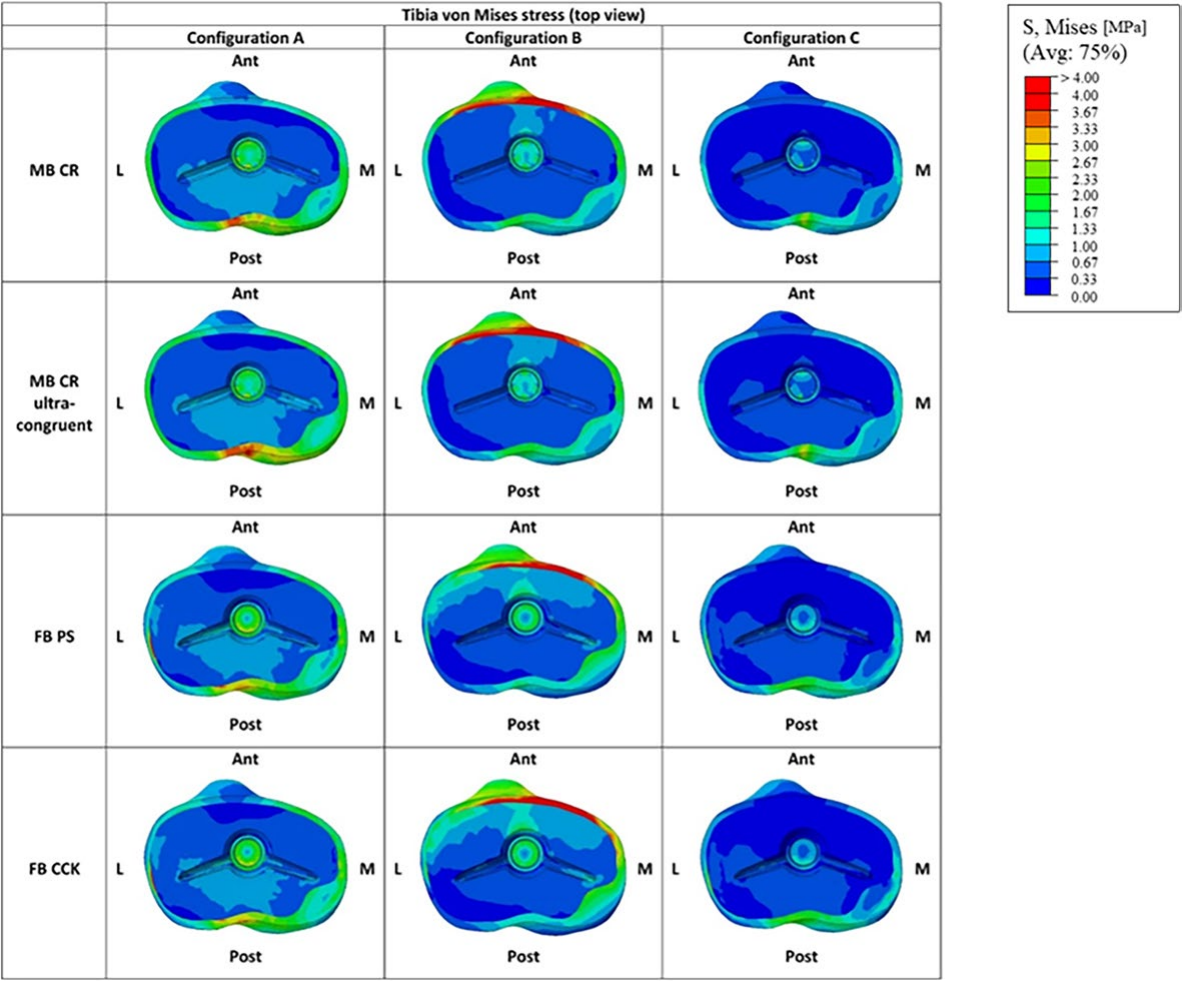
Graphic overview of the von Mises stresses in the tibial bone interface for all the considered models in the three studied configurations. (M = medial, L = lateral, Ant = anterior, Post = posterior)

Figure 10 reports the qualitative overview of the average von Mises stresses in the tibial bone interface for all the considered models in the three studied configurations. The first relevant difference in the tibial stress distribution was observed between the MB and FB models. The stress distribution in the proximal tibia was relatively homogeneous for all the models and was mainly located on the tibial cortical bone. In the configuration B, the stress concentration was located anteriorly in the insert (Fig. 9), and consequently also in the tibia this concentration was found in the anterior part (Fig. 10). In configuration C, instead, the stress concentration was located posteriorly in the insert (Fig. 9), and again consequently the same was found in the tibia (Fig. 10).

**Fig. 11:**
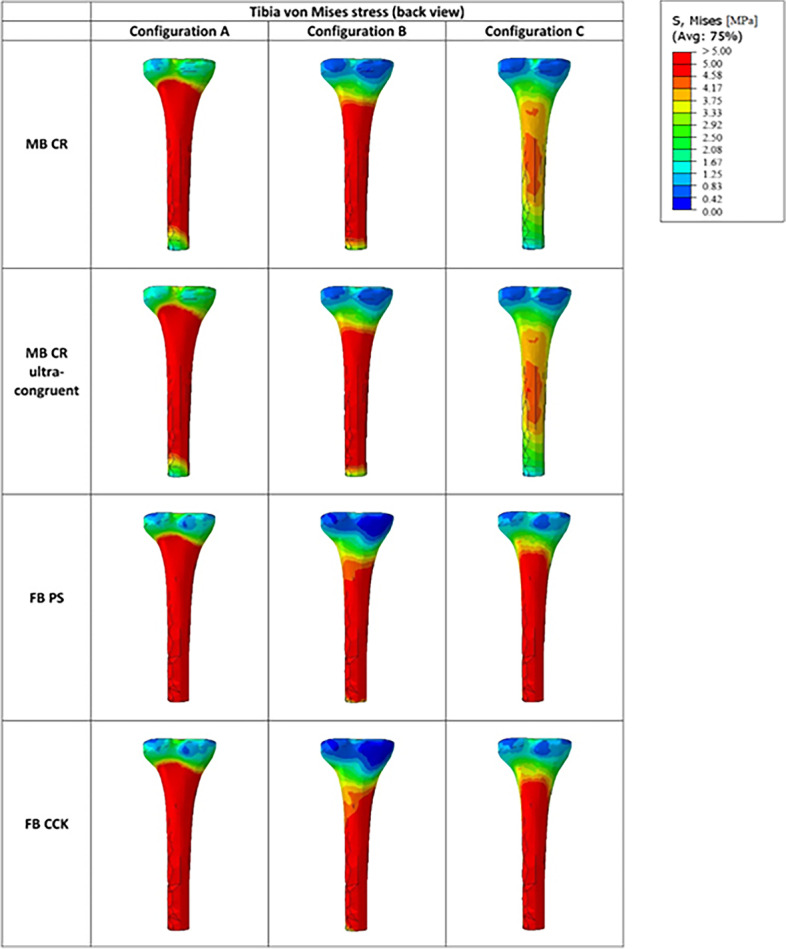
Qualitative overview of the von Mises stress along the tibial bone for all the considered models in the three studied configurations (posterior view)

Figure 11 represents the qualitative overview of the average von Mises stresses along the tibial bone, showing that this distribution bone varied from MB to FB models mostly in configuration C.

Tables 3 and 4 report the average medial and lateral von Mises stresses respectively in the proximal and distal regions of the tibial cortical bone. As shown by those tables, the distal region was usually subjected to higher tibial stress compared to the proximal one, with the stress being almost twice as high as the ones found in the proximal zone for any model in every configuration.

**Table 3:** Average medial and lateral von Mises stresses (MPa) in the proximal region of the cortical tibial bone

	Average von Mises Stresses (MPa)					
	**A**		**B**		**C**	
	**M**	**L**	**M**	**L**	**M**	**L**
MB CR	1.70	1.70	1.81	1.69	0.89	0.82
MB CR Ultra-congruent	1.89	1.80	1.88	1.73	1.00	0.88
FB PS	1.64	1.34	1.76	1.43	0.97	0.87
FB CCK	1.66	1.35	1.94	1.52	0.99	0.86

**Table 4:** Average medial and lateral von Mises stresses (MPa) in the distal region of the cortical tibial bone

	Average von Mises Stresses (MPa)					
	**A**		**B**		**C**	
	**M**	**L**	**M**	**L**	**M**	**L**
MB CR	3.73	3.06	3.52	2.79	1.77	1.40
MB CR Ultracongruent	3.80	3.05	3.53	2.82	1.81	1.40
FB PS	3.75	3.21	3.08	2.54	1.97	1.90
FB CCK	3.68	3.07	3.36	2.73	1.96	1.83

The MB CR design with a standard insert still induced higher von Mises stresses in the proximal and distal regions compared to the FB models, but only in the configuration A and B as this was not true for configuration C.

In configuration A, the proximal tibial stress for the MB CR with the standard insert was 2–4% higher in the medial region and 26–27% higher in the lateral region when compared to the tibial stresses of the FB designs. In configuration C, on the opposite, it was the proximal tibial stresses for the FB designs which were almost 9–11% higher in the medial region and 5–6% higher in the lateral region compared to the MB CR design with the standard insert.

When looking at the medial and lateral tibial stress distribution reported in Tables 3 and 4, it can be seen that the stress in the medial side was slightly higher compared to the lateral one in both proximal and distal regions of the tibial bone, for all the models. In the proximal region, the M/L distribution for both mobile-bearing models varied slightly according to the studied configuration, while it was mostly of 55/45 for both fixed-bearing models in any configuration. In the distal region, the M/L distribution was mostly 55/45 for both mobile-bearing and fixed-bearing designs in all the configurations.

## Discussion

In this study, four knee prostheses from a single family of products, each one characterized by a different constraint level, were analyzed via the finite element method in order to compare their performances in terms of contact areas as well as von Mises tibial stress in the bone and in the insert.

From the results obtained with the simulations, it emerged that the mobile bearing inserts tend to be more compliant in the anterior part, exhibiting a higher contact area in this latter.

Innocenti [9] reported the same tendency regarding the ultra-congruent insert contact area with its value in each configuration being significantly higher than those of the fixed bearings. Thus, the surface of the MB ultra-congruent insert is designed to be more compliant and therefore have a maximal contact between the femoral component and the insert.

Shiramizu *et al*. [40] highlighted a similar contact area for the FB PS insert in the first configuration with two prostheses coming from different manufacturers respectively: the NexGen LPS-flex fixed and the JOURNEY, with slightly different contact areas in the last two configurations. In this study, those differences thus resulted from all the knee models being analyzed at the different angles of flexion under the same loading condition: a vertical axial load of 3600 N. In addition, differences in the manufacturing design features also influenced the outcome for the contact areas.

Hofer *et al*. [41] reported the same trend for the contact areas with higher values for the CR prosthesis compared to the PS prosthesis. Besides, this study showed higher medial contact areas compared to the lateral ones for the CR prosthesis. This trend was, however, not observed for the PS prosthesis. Nonetheless, this study only investigated one configuration similar to ours: a squatting position at 90° of flexion.

The results of the present study showed that the distribution of the contact areas was significantly different for the mobile and fixed-bearing models in the different loading conditions, but it remains in an acceptable range. It is finally to be highlighted that the different models analyzed exhibited a symmetrical medial and lateral distribution of the contact areas, which is not always common among all the currently available prostheses (which followed the approach of the "medial pivot" design) [42].

In addition, the main relevant difference in the average tibial bone stresses was previously observed between the mobile and fixed-bearing models. The proximal and distal tibial bone stresses obtained with the different designs were similar to the ones obtained in other studies [28, 29]. Besides, by increasing the level of constraint, higher stress values were observed along the tibial bone as demonstrated in the literature by the comparison between a conventional and semi-constrained PS [13, 43].

Sathasivam and Walker [44] suggested that increased frontal plane conformity reduced subsurface stresses. However, it was not demonstrated in this study as the difference in the resulting tibial stresses between the more conforming prostheses and the others were not important enough.

Considering the results obtained and the comparison among the different models reported here, the most important finding that can be highlighted concerns the ultra-congruent mobile bearing model: This design indeed appeared to be the one able to provide the highest congruency in terms of the contact area when compared to the other prostheses analyzed, and these higher values guarantee, therefore, an overall more homogeneous stress distribution in the insert. These results, together with the literature [9], suggest thus that this design would be able to provide the best performance in terms of kinetics, in  the case that the level of constraint was suitable for the patient involved (in terms of soft tissue configuration present).

It is then remarkable to note that, regardless of the studied configuration, the distribution of the stress in the different regions of interest of the tibia (proximal and distal) was not remarkably different among all the models. Changing the prosthetic implant would therefore not induce a big variation in the tibial stress distribution. However, it would remarkably change the distribution of stress at the interface between the prosthetic components (tibial insert and femoral component) and therefore this factor is the main one to take into consideration in the decision-making process.

### Limitations

There are various limitations associated with this study, mainly related to assumptions made during the implementation of the FE models.

Soft tissues (such as the muscles or some ligaments) were not incorporated into the different models, but their contribution was nonetheless considered as an influence on the loading conditions applied [35]. Another assumption was to simplify the collateral ligaments by modeling them as beams. This is, however, a common approach in the literature and it can be found in previously validated ligaments models [9, 24–26, 31, 34, 45]. Therefore the validity of this study is not affected. All the organic material models (bony structures as well as the soft tissues) were further assumed to be linear elastic and homogeneous, although it is well-known that the cortical and cancellous bone present spatial inhomogeneity in their properties. However, such assumption, in the finite element approach for this kind of studies and for this reason, was considered acceptable [9, 25, 26, 29, 34]. Another simplification present in the study was to consider the behavior of the polyethylene as linear elastic, without taking into account the plastic region. Therefore, this approximation led to an overestimation of the local value of the polyethylene stress. However, this overestimation served a further purpose as it allowed for analysis of the eventual worst-case scenario and the results obtained showed that in no case were the critical stress values reached. It also should be considered that the goal of this study was to provide a comparison between the different studied models using the same approach [9, 25, 26], and, therefore, the fact that the same approximation is done for every model does not present an issue. The next limitation lies in the geometries used to describe the different structures: The bone models used, indeed, did not take into consideration any variation of the bone anatomy and bone deformity that could alter the final TKA outcome [9, 34, 46, 47], but this ideal approach is largely used for finite element modeling in the biomechanical field [24, 26, 48, 49] and, therefore, the study on the effect of deformities can be seen as an eventual follow-up research project. Furthermore, the list of prostheses analyzed included only one family of products, and therefore they were not comprehensive and not representative of the whole models available. However, this choice allowed for comparison of the influence of constraint of various levels only and not involving any other design features, thus representing a viable way to perform the study.

Of note, the patello-femoral joint was not included in the model, since it was not the focus of the analysis. This choice was made by taking into account the fact that the forces involved in this joint in the configurations analyzed were negligible compared to the ones involved in the considered regions of interest. Furthermore, this choice was made in agreement with previously validated models addressing similar configurations [9, 24, 28].

Finally, the positioning and the alignment of the prosthetic components were assumed to be ideal during the simulations, ignoring the possible postoperative misalignments occurring during TKA surgeries [9, 50]. Also in this case, since the main aim was to conduct a comparative study, the fact that all the models were ideally implanted should not be considered as an issue.

## Conclusion

This study investigated the biomechanics of four prostheses with different levels of constraint in total knee arthroplasty (respectively MB CR with a standard insert, MB CR with an ultra-congruent insert, FB PS and FB CCK) in three different configurations representing the typical boundary conditions taken by the knee joint during daily activities. Results demonstrated that the level of constraint influenced the femoral-insert interface, with the MB CR implant providing the highest polyethylene contact areas compared to the other prosthetic models, consequently leading to benefits on both implant survival and performance. On the contrary, bone stress appeared not to be significantly influenced by the changes in constraint levels.

## Supplementary Information

**Additional file 1.**

**Additional file 2.**

**Additional file 3.**

## Data Availability

Not applicable.

## Abbreviations

MB: Mobile bearing
FB: Fixed bearing
TKA: Total knee arthroplasty
TKR: Total knee replacement
PCL: Posterior cruciate ligament
CR: Cruciate retaining
PS: Posterior stabilized
CCK: Constrained condylar knee
LCL: Lateral collateral ligament
MCL: Medial collateral ligament
aMCL: Anterior medial collateral ligament
pMCL: Posterior medial collateral ligament
A: Anterior
P: Posterior
M: Medial
L: Lateral
