# Supplementary figures and images for: Biomechanical analysis of different levels of constraint in TKA during daily activities
Source: Arthroplasty. 2023 Jan 4;5:3. doi: 10.1186/s42836-022-00157-0 (PMC9811790; doi:10.1186/s42836-022-00157-0)

1. Element Size: 5


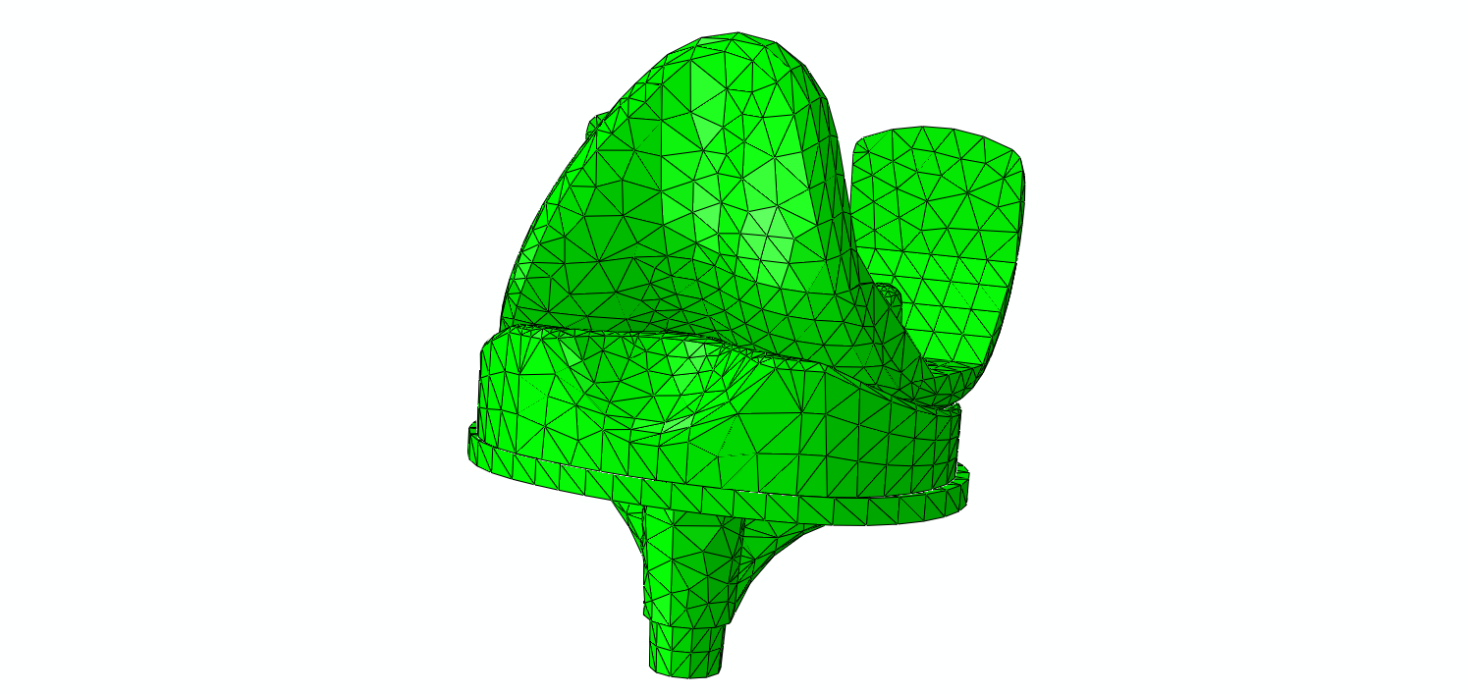


1. Element Size: 4


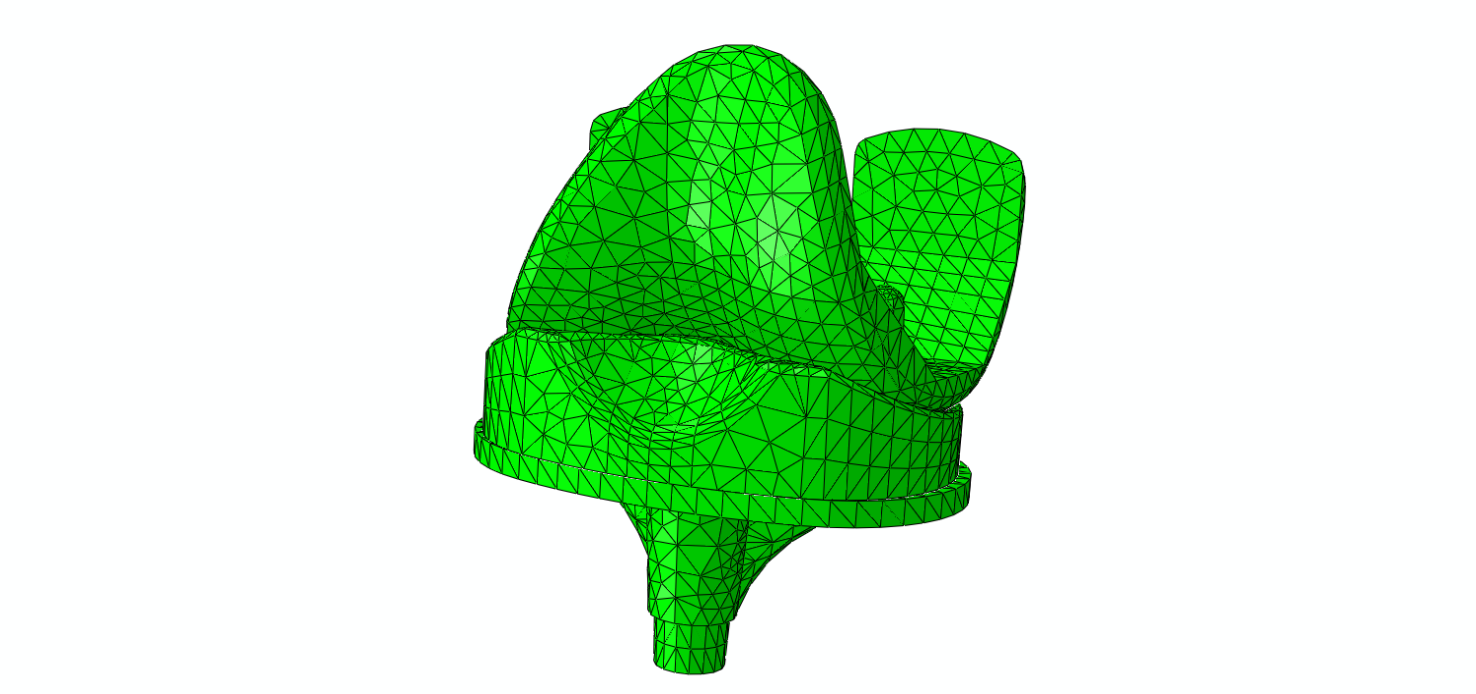


1. Element Size: 3


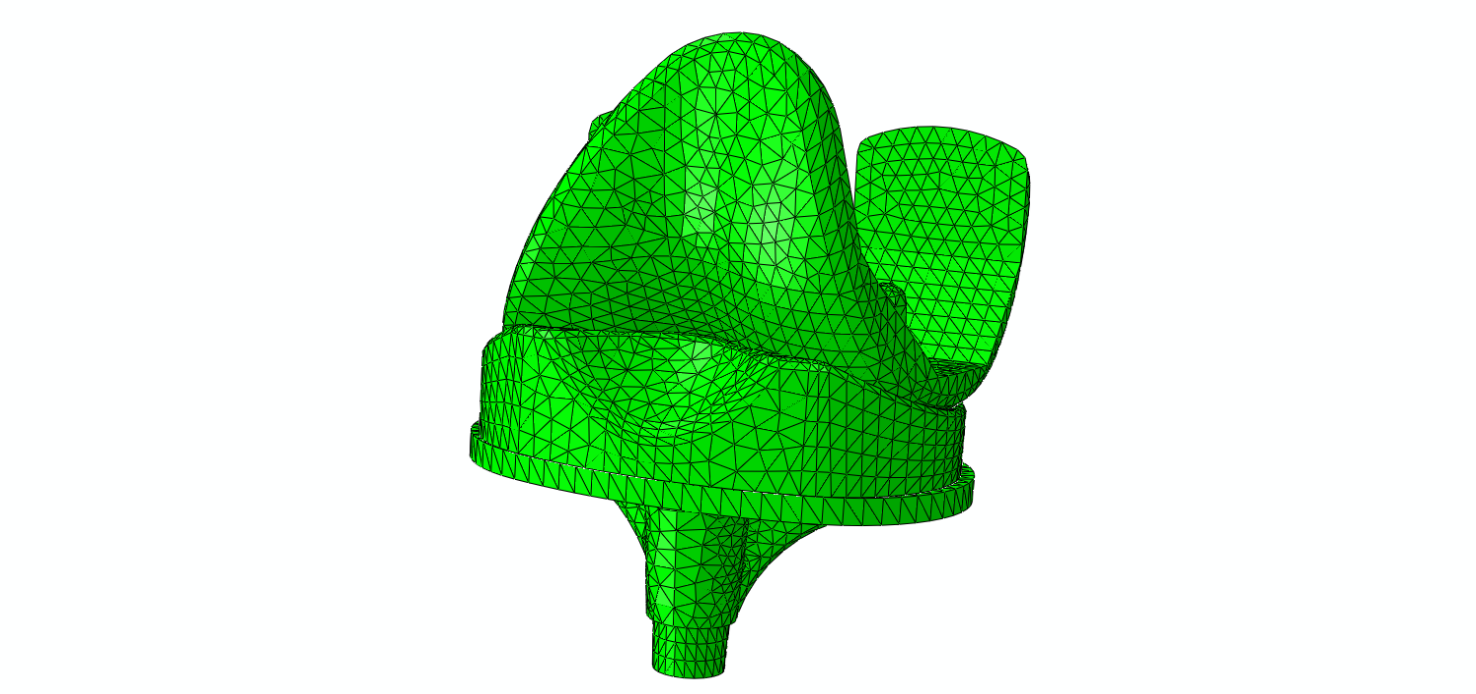


1. Element Size: 2.5


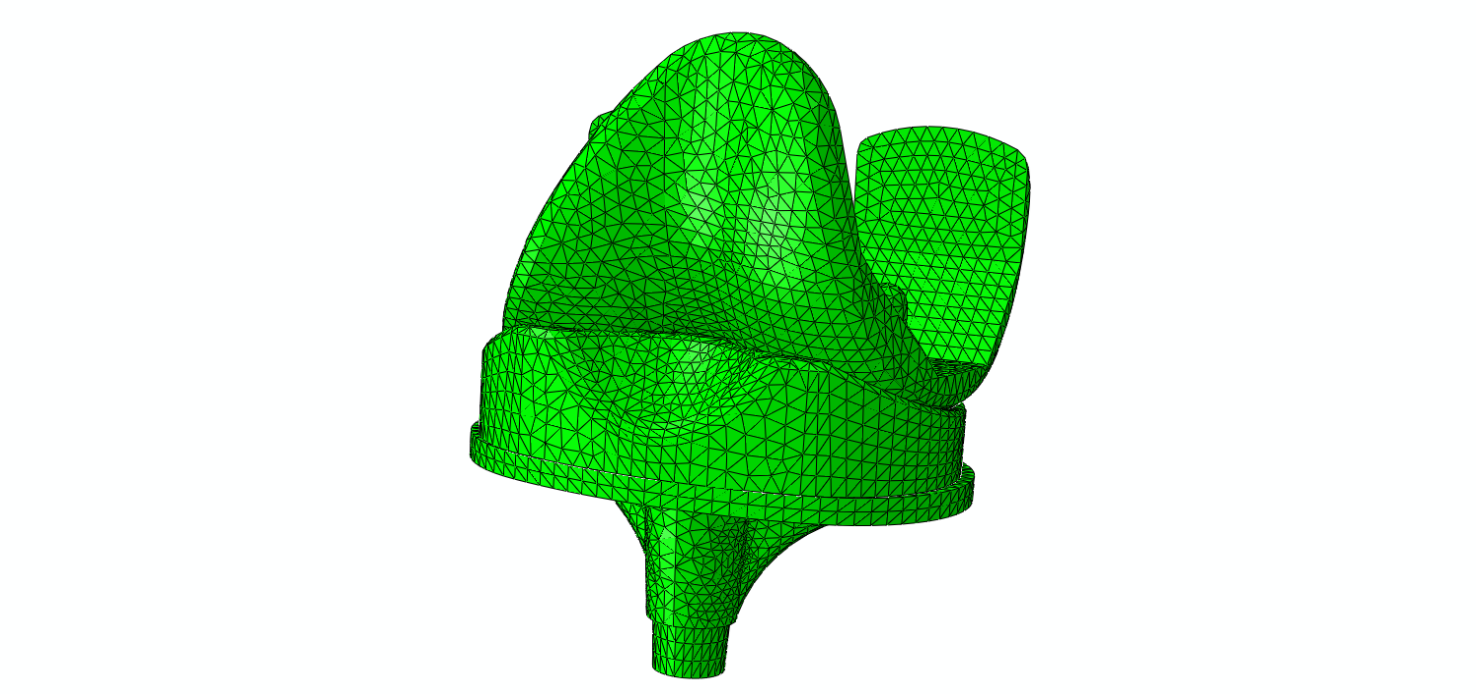


1. Element Size: 2


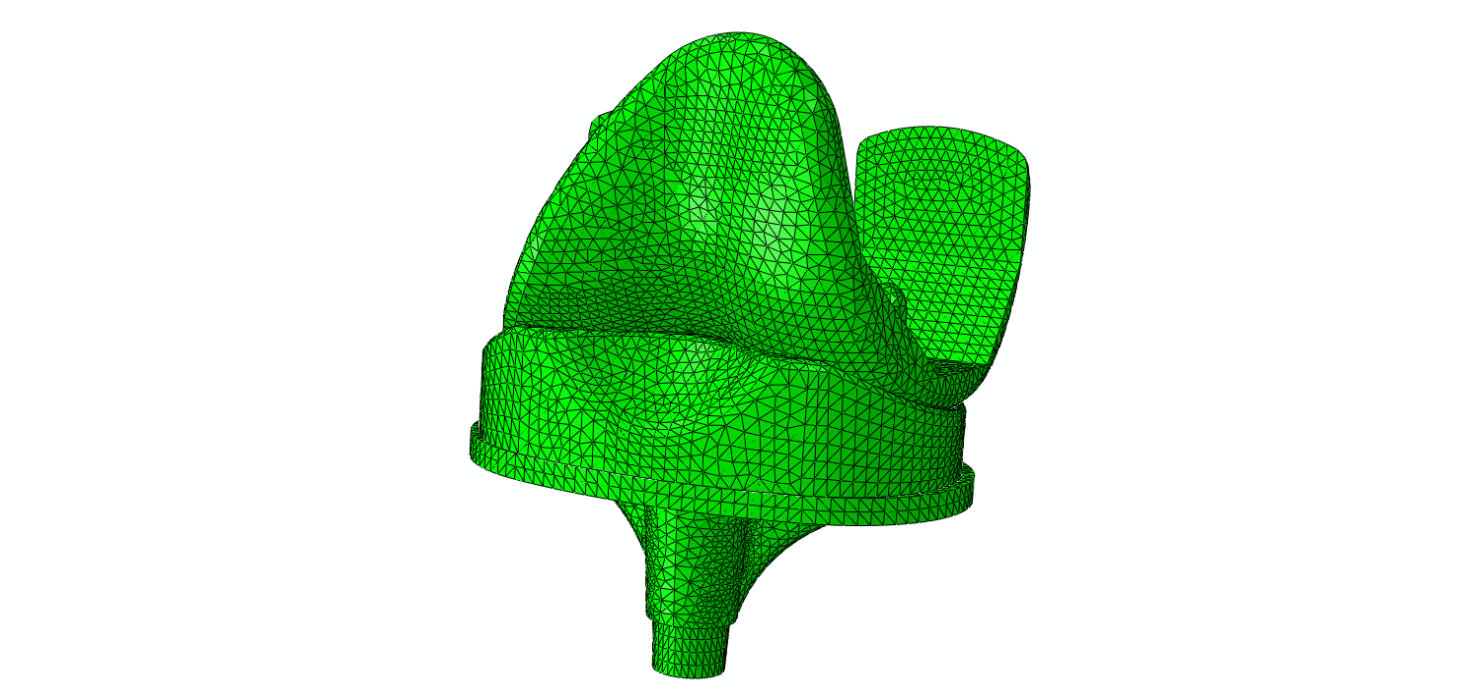


1. Element Size: 1.5


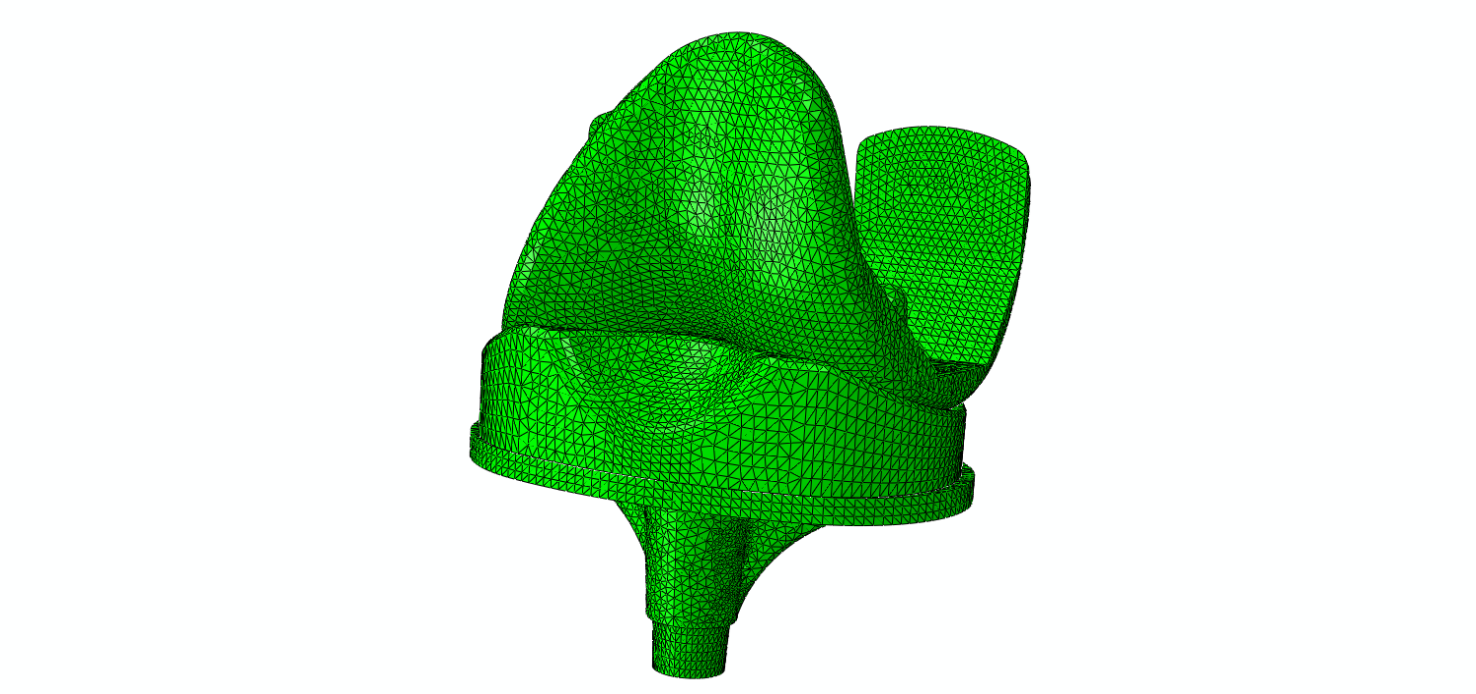

Supplement: Supplementary file 1 — Additional file 1. [file 42836_2022_157_MOESM1_ESM.docx]
