# Supplementary material for: Biomechanical analysis of different levels of constraint in TKA during daily activities
Source: Arthroplasty. 2023 Jan 4;5:3. doi: 10.1186/s42836-022-00157-0 (PMC9811790; doi:10.1186/s42836-022-00157-0)
Supplement: Supplementary file 3 — Additional file 3. [file 42836_2022_157_MOESM3_ESM.docx]

**General Contact Settings**

The interactions and setting used were chosen in according with models used in previously published studies [2, 12, 14, 19, 22, 23]: General Contact was then chosen to model the interactions involved.

Below, the definition of this typology of interaction can be found (extract from the ABAQUS user guide [https://classes.engineering.wustl.edu/2009/spring/mase5513/abaqus/docs/v6.6/books/usb/default.htm?startat=pt09ch29s03aus126.html#usb-cni-acontactgeneral]).

*The general contact algorithm generates contact forces to resist node-into-face, node-into-analytical rigid surface, and edge-into-edge contact penetrations. The primary mechanism for enforcing contact is node-to-face contact (the only mechanism used in the contact pair algorithm). If analytical rigid surfaces are present in the contact domain, the general contact algorithm also enforces node-to-analytical rigid surface contact. The general contact algorithm also considers edge-to-edge contact, which is very effective in enforcing contact that cannot be detected as penetrations of nodes into faces. For example, contact between beam segments and shell perimeter edges (see Figure) usually is detected only as edge-to-edge contact. The terminology “contact edges” refers to feature edges of surface facets (on both shells and solids) as well as to segments representing beam and truss elements. The contact edges representing beam and truss elements have a circular cross-section, regardless of the actual cross-section of the beam or truss element. The area of the circular cross-section of a beam or truss segment at a node is equal to the minimum cross-sectional area of the adjacent beam or truss elements. The radius of the cross-section is interpolated linearly over the length of the contact edge. Generally, the radius of the contact edge and the radius of the cross-section for a circular beam or truss element are not equivalent. When the axial dimension of a beam or truss element is large compared to the element section radius, the contact radius is close to the section radius over the length of the contact edge. Shell element edges reflect the shell thickness in the normal direction and do not extend past the perimeter (similar to shell nodes and facets). Some numerical rounding of features occurs for both node-to-facet and edge-to-edge contact.*

**Figure:** General contact domain, including edge-to-edge contact.


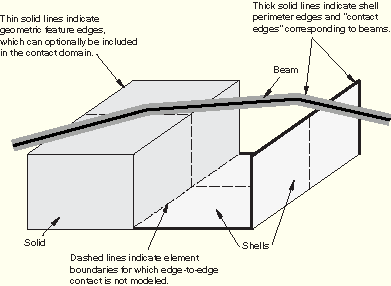


*By default, when a surface is used in a general contact interaction, all applicable facets, analytical rigid surfaces, nodes, perimeter edges, and beam and truss segments are included in the contact definition. You can control which feature edges are considered for edge-to-edge contact, as discussed in “Surface properties for general contact,” Section 29.3.2. Geometric feature edges and perimeter edges do not have to be included explicitly in a surface definition (by using edge identifiers) for them to be considered for edge-to-edge contact.*

*In edge-to-edge contact the surface around each edge is approximated as a cylinder. To model contact between edges that are not cylindrical in shape, surface elements can be attached to the edge nodes using surface-based tie constraints and node-to-face contact can be defined between the surface elements (see “Surface elements,” Section 26.7.1). This technique is useful for modeling geometric details important to the contact definition that are not modeled with the underlying element geometry. Surface elements can also be defined around shell elements in which ABAQUS has reduced the contact thickness (i.e., if the thickness exceeds the surface facet edge lengths or diagonal lengths) so that the true surface thickness can be modeled. However, using surface elements with general contact requires a physically reasonable mass to be associated with the surface element nodes, and care must be taken not to alter the bulk mass properties when transferring mass to the surface elements from the underlying elements.*

*Two-dimensional surfaces cannot be used with the general contact algorithm.*

Addressing in detail the model involved, the following specifications were implemented:

**GENERAL CONTACT**

**Contact Domain**:

-All* with self

-Excluded surface pairs : 1 item (femoral external surface – insert external surface)

- Includes all exterior faces and edges. It excludes analytical rigid structures, beam segments and reference points.

**Attribute Assignments:**

“Contact Properties” were attributed accordingly to what reported in the main manuscript.

No additional Attribute was defined in terms of “Surface Properties” or “Contact Formulation”.
